# FAMILY DYNAMICS IN CARING FOR PEOPLE WITH DEMENTIA DURING THE COVID-19 PANDEMIC

**DOI:** 10.1093/geroni/igad104.0347

**Published:** 2023-12-21

**Authors:** HwaJung Choi, Amanda Leggett

**Affiliations:** University of Michigan, Ann Arbor, Michigan, United States; Wayne State University, Detroit, Michigan, United States

## Abstract

A nuanced understanding of family care dynamics (e.g., change in care availability, care network, and care involvement) is important to assess the coping mechanism in dementia care during the pandemic. We conducted an explanatory sequential mixed methods study. We examined 300 spouses and adult children of people with dementia (PWD) from the National Health and Aging Trend -- Family and Friend, surveyed for July 2020-Feb 2021 (Quantitative analysis), and conducted qualitative analyses based on one-to-one in-depth interviews with an independent sample of 40 family members of PwD (Jan-Dec 2022). Fifty-five percent lived with PwD at the time of the interview (7% moved in with PwD during the pandemic). 35% of the non-coresident family members had fewer in-person contacts with PwD during vs. before the pandemic. More than one in ten of the sample persons lost their jobs during the pandemic. Overall, family members were more likely to provide personal care and instrumental help for PwD during vs. before the pandemic. Caregiving however is more concentrated on coresident family members during the pandemic as in-person visits were limited. Flexible work situations during the pandemic were valued as they facilitated caregiving. Family members who were not directly involved in providing personal care for PwD provided emotional and instrumental support for the primary caregiver. Findings from the study will provide valuable insights into the family dynamics in dementia care during the COVID-19 pandemic, which would inform the development of intervention programs for mitigating the adverse impacts of the pandemic and beyond.

